# Multifunctional Ground Control Points with a Wireless Network for Communication with a UAV

**DOI:** 10.3390/s19132852

**Published:** 2019-06-27

**Authors:** Xiongzhe Han, J. Alex Thomasson, Yang Xiang, Hussein Gharakhani, Pappu K. Yadav, William L. Rooney

**Affiliations:** 1 Department of Biological and Agricultural Engineering, Texas A&M University, College Station, TX 77843, USA; 2 College of Engineering, Hunan Agricultural University, Changsha, Hunan 410128, China; 3 Department of Soil and Crop Sciences, Texas A&M University, College Station, TX 77843, USA

**Keywords:** ground control points, unmanned aerial vehicle, wireless network, radiometric calibration, height calibration

## Abstract

Ground control points (GCPs) are commonly used for georeferencing in remote sensing. Precise position measurement of the GCPs typically requires careful ground surveying, which is time-consuming and labor-intensive and thus excessively costly if it needs to be repeated multiple times in a season. A system of multifunctional GCPs and a wireless network for communication with an unmanned aerial vehicle (UAV) was developed to improve the speed of GCP setup and provide GCP data collection in real-time during the flight. While testing the system, a single-board computer on a fixed-wing UAV used in the study successfully recorded position data from all the GCPs during the flight. The multifunctional GCPs were also tested for use as references for calibration of reflectance and height for field objects like crops. The test of radiometric calibration resulted in an average reflectance error of 2.0% and a strong relationship (R^2^ = 0.99) between UAV-based estimates and ground reflectance. Furthermore, the average height difference between UAV-based height estimates and ground measurements was within 10 cm.

## 1. Introduction

Unmanned aerial vehicles (UAVs) are being used in agricultural research to collect images for various tasks including biomass monitoring, crop yield estimation, disease detection, and water status estimation [1,2,3]. In transitioning, for these type of data to on-farm use, high accuracy is critical to reliable decision-making. Accurate positions in three-dimensional (3D) space require georeferencing of the UAV images [4,5]. Other requirements for high accuracy can include calibrating radiometric reflectance from multispectral-camera images and object height from digital surface models (DSMs).

Ground control points (GCPs) with precisely measured positions are needed to improve the geometric accuracy and quality of digital terrain models (DTMs), DSMs, and orthomosaic maps [6,7,8] unless a direct georeferencing technique is used [9]. Such GCPs are commonly used for georeferencing and are designed to be clearly visible and large enough in images so that a center point can be accurately and precisely marked from the flight altitude. It is crucial that the UAV has an unobstructed view of the GCPs from the sky and that shadows from close-by objects are avoided. A precise location measurement based on real-time kinematic (RTK) or post processing kinematic (PPK) global positioning system (GPS) is required for optimal use of GCPs. In addition, GCPs should be spread across the ground area of the flight zone and should cover both high and low elevations [10]. 

Precise position measurement commonly requires careful ground surveying, which is time-consuming and labor-intensive and thus excessively costly if it needs to be repeated multiple times in a growing season. The commercially available AeroPoint GCP (Propeller Aero, Sydney, NSW, Australia) is reportedly simple to use, portable, reusable, and commonly used (e.g., in surveys for mining) to record its position during UAV flights [11]. Multiple Aeropoints can be used collaboratively to increase the accuracy of each unit in the field. Researchers in agricultural and forestry remote sensing have used AeroPoints as geographic references [12,13], but these GCPs need to be active for at least 45 min to record enough position data to be survey-grade accurate, and they would typically need to be distributed across the field immediately prior to each flight mission. Thus, their use may not be practical in agricultural-production situations. Moreover, the GCPs utilized in agricultural remote sensing research have typically been used as single-purpose devices, such as for only position measurement, but they can potentially also serve as references for other measurements.

While agricultural remote sensing requires accurate position data [14,15], it may also require accurate radiometric and plant-height data. Radiometric calibration is essential to generate consistent remotely sensed reflectance imagery under a wide variety of environmental conditions [16,17]. Height calibration can also be used to improve the accuracy of plant height estimation from a DSM [18].

For high geometric accuracy, the 3D coordinates of GCPs are typically surveyed with a GPS receiver working in PPK or RTK mode with a base station on a geodesic pillar located closer than one kilometer from the studied surfaces [19,20]. At least five GCPs are recommended to maximize accuracy, but further increasing the number of GCPs leads to even higher accuracy in point clouds, orthomosaics, and DSMs [21,22]. If multiple GCPs have to be positioned in the field, particularly if this process must occur multiple times during a growing season, manually surveying their positions is burdensome and most likely impractical on farms. However, if GCPs could not only measure their own position as Aeropoints do, but do so quickly and also communicate the data to an over-flying UAV, GCP position could potentially be embedded in the image metadata, and the efficiency of data processing could be significantly improved.

In recent research [23], software has been developed to use GCPs in UAV-based image mosaics to automatically correct them for radiometric fidelity. The software recognizes reflectance targets on the GCPs, extracts digital numbers from them, and calibrates the entire mosaic. This software can replace the conventional semi-manual methods that are typically required to process the raw image data after the flight. However, additional automated systems are needed to make UAV-based remote sensing practical on-farm.

Communications in agricultural fields are a relatively new phenomenon, but wireless sensor networks are becoming commonplace for applications like weather monitoring and irrigation management [24,25,26]. Among other variables, the sensors may collect data on humidity, temperature, and soil moisture, all significant environmental parameters for plant growth [27,28,29,30]. The design of an agricultural sensor network should ensure the availability and authenticity of the data. Certain network architectures, mechanisms, and algorithms have been developed to ensure high-quality communication. Individual networked sensors typically serve as nodes in the network, and this design increases transmission efficiency for precision-agriculture applications [31,32,33] and can ensure reliability in communication. While wireless sensor networks are becoming more common in production agriculture, they have not been researched for real-time communication between UAVs and ground-based references to enable acquisition of reference information for high-quality imagery for precision agriculture.

As previously mentioned, there are limitations to use GCPs in agricultural remote sensing with UAVs. Current research gaps include a lack of utilizing GCPs for other referencing needs like radiometric and plant-height calibrations as well as a lack of study on wireless networks that could link UAVs and GCPs in real time for automated reference data collection.

The overall goal of this research involves developing an autonomous GCP system including a wireless network for position-data collection and communication with an UAV. Such a system could improve the speed of GCP setup and provide data collection advantages that have broad application in agriculture and environmental monitoring. Specific objectives of this study were (1) to design, build, and test portable GCPs for multiple referencing purposes (georeferencing, radiometric calibration, and height calibration); (2) to develop and test a wireless network for automatic GCP position data collection and automatic communication with a UAV; and (3) to evaluate the system in terms of quality of calibration for reflectance and plant-height maps.

## 2. Materials and Methods

### 2.1. System Architecture and Principles

**Figure 1:**
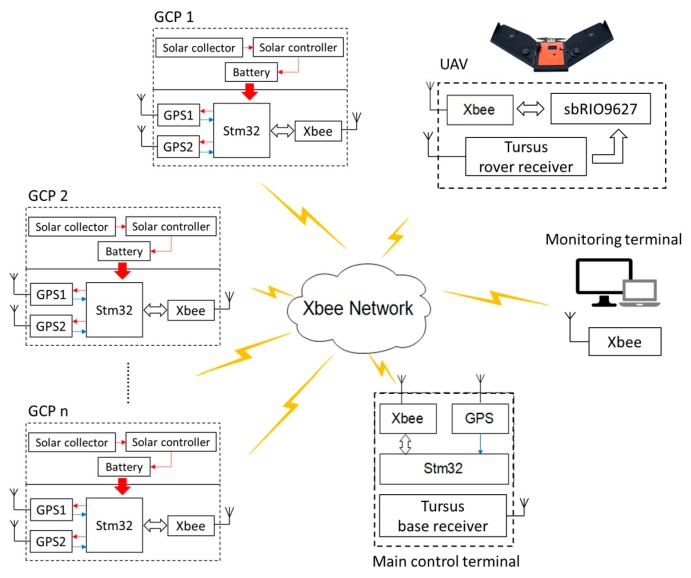
Overall schematic diagram of the wireless system network.

**Figure 2:**
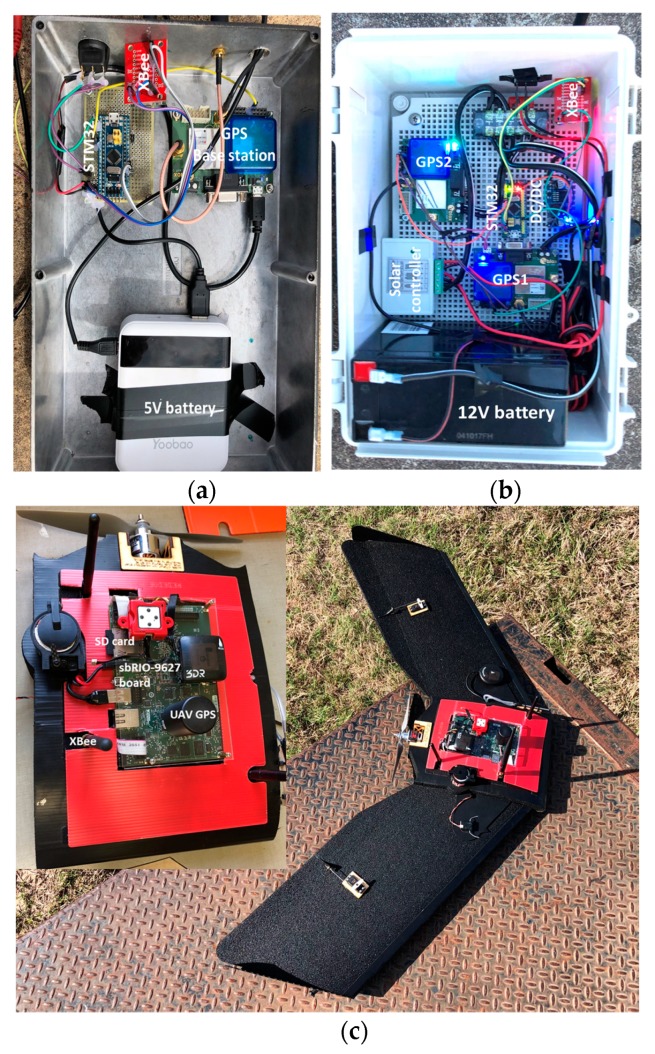
View of (**a**) the main control terminal, (**b**) the integrated ground control point (GCP) controller and (**c**) the embedded control board on the fixed-wing unmanned aerial vehicle (UAV).

The entire GCP system (Figure 1) includes a GPS base-station with a main control terminal serving as a central coordinator, a laptop running a custom user interface to serve as a monitoring terminal for the experiment, several GCPs carrying subordinate GPS receivers, a fixed-wing UAV, and a wireless network with multiple hardware and software components. The main control terminal (Figure 2a) included a low-cost GPS module (C94-M8P RTK-GPS; Ublox, Thalwil, Switzerland) serving in base mode, an STM32 microcontroller (STM32F103C8T6; STMicroelectronics, Geneva, Switzerland), and an XBee radio transceiver (XBP9B-DMST-002; Digi International, Minnetonka, MN, USA). Differential-correction signals collected by the base station were transmitted through a universal asynchronous receiver/transmitter (UART) serial communication interface to the STM32 microcontroller, which then broadcasted them to the GCPs through the XBee radio transceiver.

Each GCP included two of the low-cost GPS modules acting in rover mode, an STM32 micro controller, a solar collector and controller (L02P25X-CB-1; ECO-WORTHY, Taiwan, China), a 12 V battery, and an XBee radio transceiver (Figure 2b). The GPS modules were used to measure horizontal and vertical positions of the GPS antennas at 10 Hz with a baud rate of 19,200 bps, and having two GPS modules on each GCP enabled calculation of the angle of attitude of each GCP in the field. The angle of attitude is important if one is attempting to use software to automatically identify a GCP in the image data. The STM32 microcontroller was a relatively inexpensive processing chip for communicating with the main control terminal through the Xbee radio transceiver. The differential-correction signals produced by the main control terminal were used to correct the output of the two GPS modules on each GCP to improve positioning accuracy in real time. The solar collector and controller were used to charge the 12 V battery, which powered all the hardware on each GCP.

The corrected GPS data on the GCPs were sent to the main control terminal through the Xbee radio transceiver and comprised latitude, longitude, and elevation of each GPS antenna. The final data package, including all GCP information assembled as a text file by the main control terminal, was transferred through the Xbee module to an SD card in an embedded single-board computer (sbRIO-9627; National Instrument, Austin, TX, USA) on an airborne UAV and also to a laptop used for monitoring. The fixed-wing UAV used in this research (Tuffwing Mapper; Tuffwing LLC, Boerne, TX, USA; Figure 2c) was equipped with a more-expensive GPS receiver (BX305; Tersus Gnss Inc., Shanghai; China), which received a correction signal from a similar base receiver on the main control terminal. Thus, for purposes of this experiment, the main control terminal housed two different types of GPS receivers acting as base stations for the different types of rover GPS receivers on the GCPs and the UAV. It should be noted that the simplest conceivable version of the GCP system would have neither a monitoring terminal nor a base station, assuming a local differential correction signal was available. In such a case the remaining functions of the main control terminal could be moved to the single-board computer on the UAV.

### 2.2. GCP Structure

**Figure 3:**
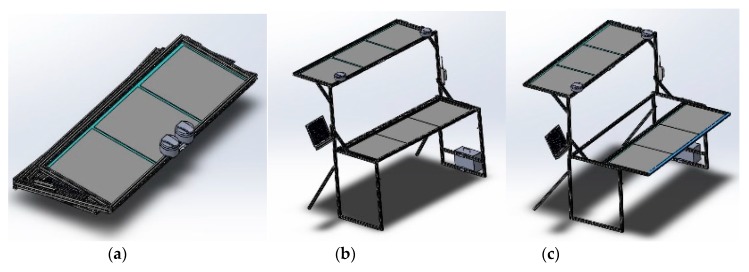
Structure of the aluminum frame of the multi-functional GCP which is (**a**) portable, (**b**) two level layers and (**c**) extendible for the bottom panel.

Portable and foldable (Figure 3a) GCPs were designed with aluminum frames to minimize weight while maintaining strength and functionality. The structure of the GCPs was to be an improvement over an earlier version constructed with wooden frames that were affixed to the ground to act as a semi-permanent calibration system in a previous study [18]. Each new GCP was to have two layers with three 24-inch square reflectance references (dark, medium, and light gray) installed on each layer (Figure 3b). The height from the ground to the bottom and top panels was to be 36 and 72 inches, respectively. The bottom panel was designed to be extended outward to avoid shadows cast from the upper layer onto the lower layer (Figure 3c). The weight of the GCP was 20 kg without any controller components. Seven of these GCPs were constructed and used in this research.

### 2.3. Data Processing Strategy

The embedded radio modem on the low-cost GPS modules had a limited number of channels that constrained the ability to connect multiple rover GPS modules with the common base station. Thus, external power of 3.3 VDC was applied to appropriate ports on the GPS modules to turn off the radio modems and enable external UART communication. The differential-correction data provided by the main control terminal were transferred to the STM32 microcontroller at a frequency of 1 Hz. Prior to the transmission, a marker was added at the beginning and end of the GPS sentence associated with the differential correction data for the purpose of unambiguous reception. These markers were removed by the microcontroller on each GCP before the differential-correction data were used to correct the GPS data on the GCPs.

**Figure 4:**
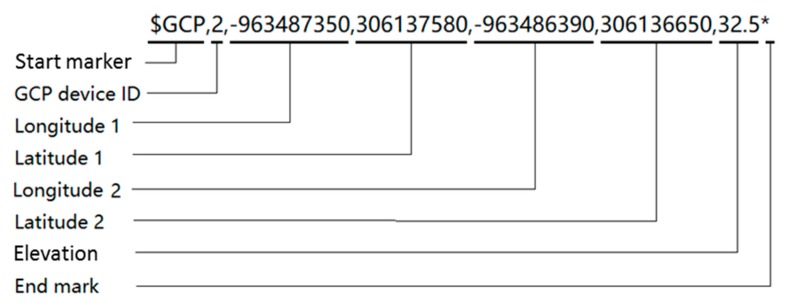
Structure of the GCP data transferred from the main controller to the UAV.

The main control terminal was configured as the central coordinator of the wireless network to avoid communication conflicts between the GCPs and the UAV. Once the receiving status of the GPS modules was fixed at centimeter-level accuracy, usually within 2 min of start-up, each GPS data package was temporarily saved in the internal memory of the associated GCP’s STM32 microcontroller. Table 1 shows the format of a query instruction, which was transmitted in a hexadecimal string including two bytes “0x6677” for a start marker and one byte “0x88” for an end marker. The device ID in Table 1 indicates the requested target GCP among the GCPs distributed in the field, and the instruction ID is 10 H, which represents the query command for GCP position information from each GCP. When a particular GCP’s device ID was specified in the UAV’s query instruction, the GPS data package of that GCP was sent to the main control terminal in the format shown in Table 2. The data package was a 20-byte string sentence, including positions (i.e., latitude, longitude, and elevation) for the left and right sides of the GCP, with two bytes “0x6688” for a start marker (Figure 4). The GCP data sentence was formed in the main control terminal and sent to the fixed-wing UAV by encapsulating the data in the aforementioned format.

### 2.4. Field Testing

#### 2.4.1. Accuracy Assessment

**Figure 5:**
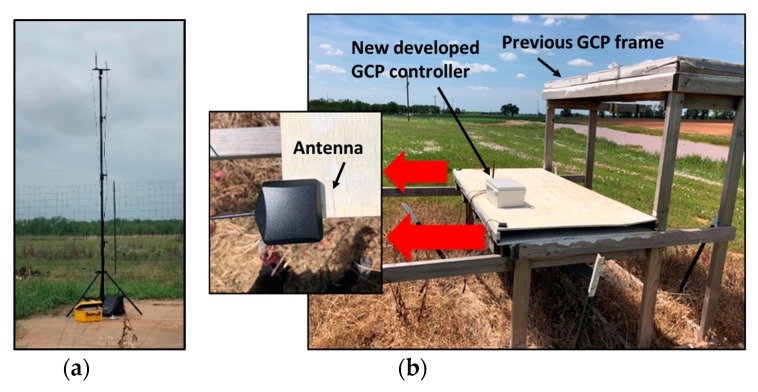
View of (**a**) the base station on the surveyed position and (**b**) the accuracy assessment test in the field.

The base station (Figure 5a) was installed precisely on a position near the test field that was part of the Online Positioning User Service maintained by National Geodetic Survey [34], which provides simplified access to high-accuracy National Spatial Reference System coordinates. Seventy GCPs (Figure 5b) were developed for the prior 2017 research season, and eight were used for a prior study [17]. Four of the 70 were used in 2018 as positional references to evaluate the accuracy of the new GCPs. The 2017 GCPs had been held in place with steel anchors since the prior season. The positional correction signal was used from base stations installed at the same National Geodetic Surveyed position in both 2017 and 2018. A Trimble R8 GNSS unit and R7 base station, with an accuracy of 1 cm + 1 ppm horizontal and 2 cm + 1 ppm vertical after post-correction, were used to collect a GPS position measurement at the front left and front right corners of the lower layer on all GCPs used in 2017. The antennas of the new GCPs were also placed on the same positional reference locations of the previous GCPs to compare the horizontal and vertical accuracies and allow observation of measurement stability in the new low-cost GCP system.

#### 2.4.2. Validation Experiment

**Figure 6:**
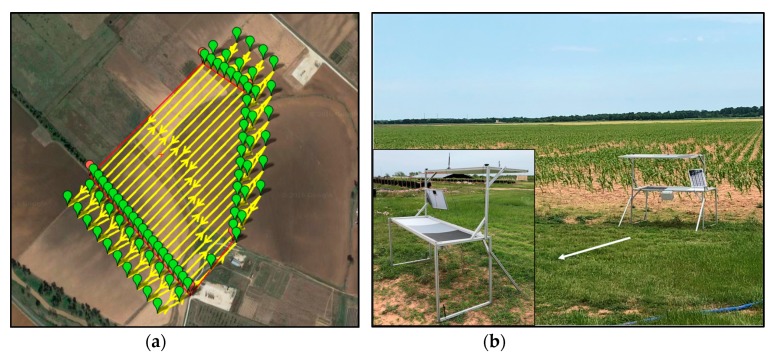
View of (**a**) the planned flight path pattern generated for the UAV and (**b**) the validation test in the field.

The Tuffwing UAV was flown with a multispectral camera (RedEdge; MicaSense, Seattle, WA, USA) at an altitude of 120 m above ground level (AGL) and a ground speed of approximately 17 m/s. Figure 6a shows the planned flight path used during flight missions, which achieved sufficient side overlap for subsequent image mosaicking. The new GCPs were distributed across a field of 0.35 km^2^ area at Texas A&M AgriLife Research farm near College Station, TX, USA. Three crop types—cotton, corn, and sorghum (Figure 6b)—were covered in this study. The wireless network was used to determine whether all GCP information could be successfully transmitted and recorded as image metadata onboard the UAV during flight, for purposes of georeferencing, radiometric calibration, and height calibration. A custom user interface program was written in Labview to run on the monitoring terminal, so that the reception status of the GCPs could be verified in real time. 

**Figure 7:**
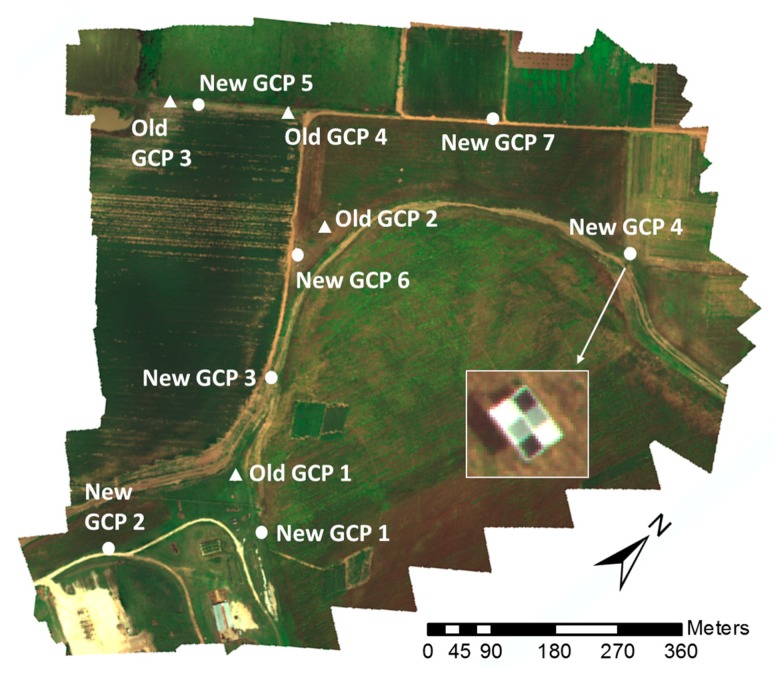
Distribution of all GCPs in the experiment field at Texas A&M AgriLife Research farm.

The seven GCPs were distributed to broadly cover the test field (Figure 7). To minimize the effects of changing illumination and atmospheric conditions on UAV images, radiometric calibration was conducted by using the reflectance references on the GCPs. Roughly 30 image pixels were extracted from each calibration reference on GCPs 1 through 5 to determine the median reflectance values for each band of the multispectral camera. Ground reflectance (%) measurements were collected from five uniformly distributed points on each calibration reference with a handheld spectrophotometer (PSR+; Spectral Evolution, Lawrence, KS, USA) within 15 min after the flight. The median reflectance values of the calibration references were obtained from the five spectral bands. A calibration equation was derived by fitting the digital numbers DNs of the images to the reflectance values of the calibration references for each band, the DNs of each band were converted to normalized reflectance values. To validate the radiometric calibration, the average reflectance error for each band in the calibrated image mosaic was calculated based on measurements of GCPs 6 and 7, which were located at different positions in the field from the five GCPs used for calibration.

Consistent with the method used in a previous study [18,35,36], height calibration of the image mosaic from each flight test was performed with a linear equation based on three points extracted from each GCP location for GCPs 1 through 5: Ground elevation immediately adjacent to the GCP, the GCP’s lower layer, and the GCP’s upper layer. The DTM had been created by using structure-from-motion on image data collected previously during bare-ground conditions. Similar to the test for radiometric calibration, the average height error in the calibrated DSM was calculated based on each level of GCPs 6 and 7.

## 3. Results and Discussion

### 3.1. GCP Physical System and Position Accuracy

**Figure 8:**
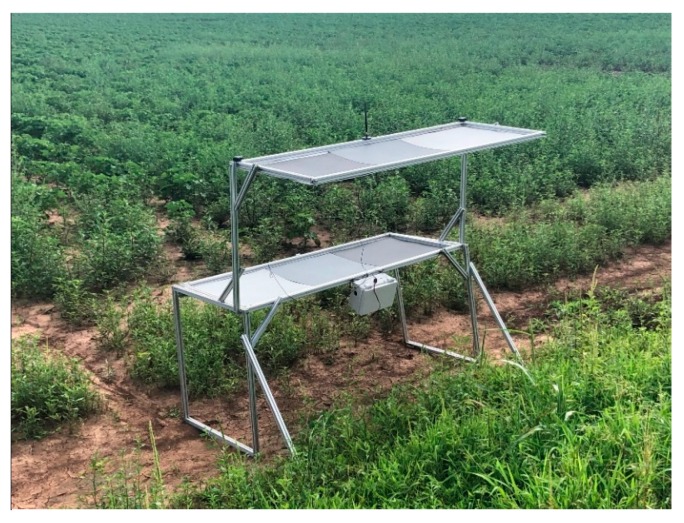
Real constructed multifunctional GCP used for georeferencing, radiometric calibration, and height calibration.

Figure 8 shows a portable, foldable, multifunctional GCP as constructed and placed in the field. The cost of materials and components of each GCP system was $480. All hardware components of each GCP were powered successfully by the battery, which was recharged by the solar collector. The time required for GCP setup by two workers was approximately 40 s, and the time spent measuring and recording GCP positions with the wireless network was approximately 2 min, compared to 30 to 60 min when placed manually, a particularly important consideration when data collection must be repeated multiple times in a season.

**Figure 9:**
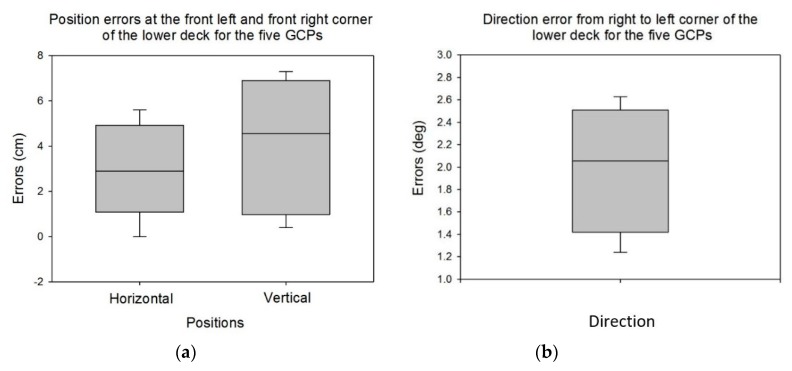
Errors of (**a**) the horizontal position, vertical position, and (**b**) direction measured with the developed GCPs system.

The results of comparing the new GCP coordinates with prior-year reference coordinates are shown in Figure 9. Position errors at the front left and front right corner of the lower layer for the four GCPs were within 5.6 cm for horizontal and 7.3 cm for vertical (Figure 9a). The direction error from right to left corner was from 1.2 to 2.6 degrees (Figure 9b). Average errors were 2.3 cm, 4.2 cm, and 1.9 degrees for horizontal position, vertical position, and direction, respectively (Table 3). These average errors indicate that the low-cost GPS units used with the GCPs in this study enable the GCPs to have potential for future UAV remote-sensing applications. In current agricultural remote-sensing research, GCPs are typically placed in the field, their positions are carefully measured, and they are manually identified in the resulting images for georeferencing of the image mosaics. The image processes are commonly implemented in commercial geographic information systems (GIS) software like ArcGIS or image-analysis software like ENVI. An automated georeferencing algorithm was developed in recent research [23] to automatically identify the GCPs in an image mosaic. Once identified, information on position, reflectance, and crop height could be readily extracted and applied for georeferencing, radiometric calibration, and height calibration, respectively. The computational efficiency of this algorithm could be vastly improved if small search zones could be defined in the initial image mosaic based on known positions of the GCPs. The lower the position error of the GCPs, the more efficient the algorithm.

### 3.2. Performance of the Wireless Network

**Figure 10:**
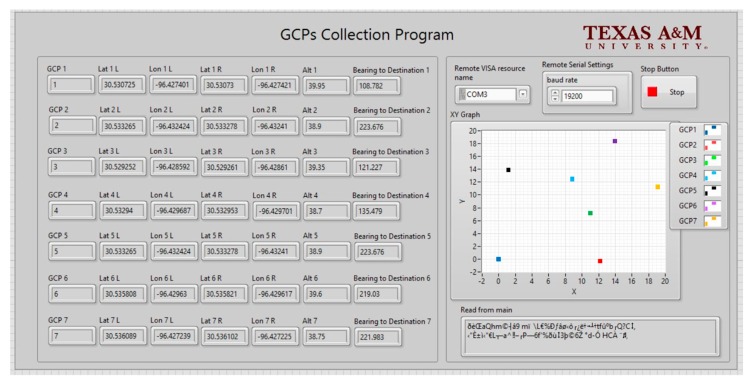
User interface of the GCPs collection program.

Figure 10 shows the user interface (UI) of the GCP data collection program, which was deployed on the monitoring terminal for observation and functioned as designed during the experiments. As shown on the UI screen, the GCP data were displayed in relation to their string format to include each GCP’s device ID, left and right side coordinates, elevation, and direction. The signals from the RTK-GPS on the fixed-wing UAV were transmitted through the wireless network to the GCP data collection program, allowing the movement of the UAV to be displayed in real time. The wireless network was highly reliable, with failure-free operation in the field test at distances between ground station and each GCP, and also between the ground station and UAV, of up to 1.6 km.

The GPS data of the seven GCPs—device ID, positions on left and right sides of the GCP, elevation, and direction—were automatically recorded in the txt file (Table 4) on the fixed-wing UAV in real time during the flight. The GCP txt files could be used to enable efficient fully automated extraction of GCP information during post-processing of images. As mentioned previously, a prior study [22] involved automating georeferencing and radiometric correction of UAV-based image mosaics. Applications requiring high-quality georeferencing of image mosaics require accurate GCP position data, and the GCPs in this study can provide such data. In future applications, the GCP data file generated in this study could be embedded into image metadata as a header file, so that georeferencing and radiometric calibration of UAV-based mosaics could be automated for higher speed, lower cost, and higher objectivity than current common manual methods.

### 3.3. Field Validation Test

Table 5 shows the radiometric calibration coefficients derived by fitting the digital number (DN) values of the images to the reflectance data of the calibration targets for each of the red, green, blue, red edge, and near infrared (NIR) bands. The results indicated that DN values could be successfully converted into reflectance data by way of linear relationships with R^2^ values in the range of 0.9622–0.9943 for all five bands. The R^2^ values of the red edge and NIR bands were clearly lower than with the other bands. However, all the radiometric calibrations indicated a strong correlation between ground reflectance and the images’ DN values. Thus, radiometric calibration can be used to minimize the effects of variation in illumination and atmospheric conditions on UAV images taken at different times [37].

**Figure 11:**
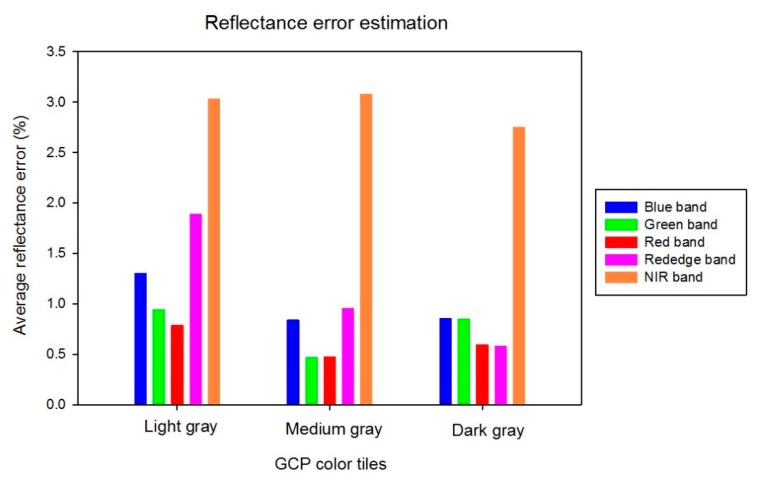
Average reflectance calibration error for each color tile and each band.

**Figure 12:**
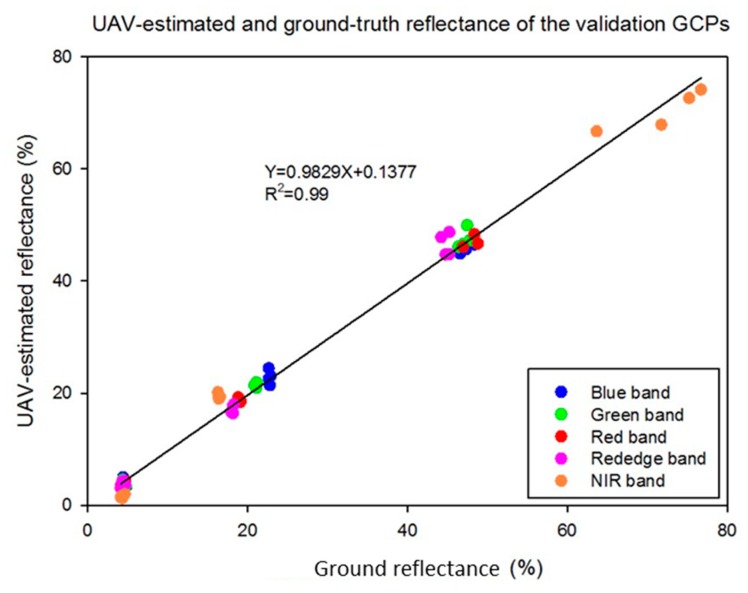
Correlations between UAV-estimated reflectance and ground reflectance for all five bands.

In terms of the red, green, and blue bands, the intercept values were similar and close to zero. However, the intercept values of the red edge (−14.2) and NIR (−20.7) bands were much higher than with the visible bands. Not surprisingly, the highest error in reflectance estimation was observed in the NIR band (Figure 11), but the maximum average error was still only 3%. A similar prior study [38] reported a maximum error of 8.0% and an average error of 2.5%, higher errors than reported here, so the numbers in this study are relatively low. However, based on the results of this study and other related field tests, it was determined that there was excessive noise in the red edge and NIR bands of the multispectral camera used in this work, but even so a maximum 3.0% reflectance error was found in the field test with the NIR band. The average reflectances of the remaining bands were within 2.0% of ground values, and usually within 1.0%. The red band had the lowest average errors at 0.78%, 0.47%, and 0.50% for the light gray, medium gray, and dark gray references, respectively. The performance of the radiometric calibration was excellent, providing for a strong relationship (R^2^ = 0.99) between UAV estimates and ground reflectance of the validation GCPs across all camera bands (Figure 12). While the calibration data came from three references of differing gray levels, it is clear that the calibration data consist of four clusters of points. The points at around 75% reflectance indicate that the NIR band on the light gray reference was significantly higher than reflectance of that reference in the other bands. The slope and offset of the regression line between UAV-based estimates of GCPs reflectance and ground measurements were 0.98 and 0.14, respectively. Therefore, it is clear that the GCP system evaluated in this study provides the potential for high-quality radiometric calibration of multispectral UAV image mosaics.

The average difference between UAV height estimates and ground measurements of the validation GCPs is shown in Table 6. Relatively low (<10 cm) average height error existed for the lower and upper GCP layers. Part of the reason for the height error is the inherent error in vertical position measurements with the RTK-GPS system, which affects the position accuracy of the GCPs used to correct the data on UAV images [3,39,40]. That vertical error determined in this study averaged 4.2 cm. Prior work [15] indicated that image pixels of known-height ground references collected from a fixed-wing UAV at 120 m AGL can be used to calibrate the DSM reasonably well for the purpose of reducing height errors. Results of the current study tend to corroborate that finding, with object height errors similar to the better results of the prior work. It should thus be possible to effectively apply the new GCPs for height calibration if they are appropriately distributed across the field.

### 3.4. Advantages and Limitations

The main advantages of the system proposed in this study are as follows: (1) That the multifunctional GCP enables image data calibration of not only position, but also reflectance and object height; (2) that the low-cost and high-accuracy GPS receivers onboard the GCP enable autonomous position and attitude measurement; and (3) that the wireless network technology enables GCP position data to be saved alongside image data onboard the UAV. Taken together these capabilities mitigate the following problems: (a) High error in position, reflectance, and plant height; (b) the labor and time required for surveying GCPs for remote sensing of crops in a large field during a growing season; and (c) the requirement for manual identification of GCPs in large image mosaics and manual extraction of their digital numbers for calibration. Even though direct georeferencing could potentially replace conventional single-purpose GCPs in some circumstances [9], the multifunctional, autonomous, ground-based system in this research provides multiple additional benefits over simply georeferencing. Some limitations of the proposed system include cost of each GCP, which would likely be a few hundred dollars (USD in 2019) on the open market. Thus, such a system is likely commercially practical only in high-value crops. Another possible concern is having two layers of references on the GCP, which tends to make the GCP large for some high-value applications like vineyards. Furthermore, modifications to current UAVs are required to enable the system to operate as designed. One point of note, however, is that the functions of the main control terminal—which was positioned on the ground in this experimental setup—could potentially be moved to the single-board computer on the UAV to simplify the ground system, and even base stations would not be required if some type of local correction signal would be commonly available.

## 4. Conclusions

A multifunctional GCP system was developed for the purposes of minimizing GCP setup time and improving efficiency in data collection, mosaicking, georeferencing, and calibration of the orthomosaic for plant reflectance and height measurements. A wireless network was developed for automatic collection of GCP position data and communication with UAV. The system enabled recording of all GCP data onboard a fixed-wing UAV in real time during a flight mission. Average errors of position measurements with the GCP receivers were 2.3 cm horizontal position, 4.2 cm vertical position, and 1.9 degrees direction, suggesting that such a GCP system with inexpensive GPS receivers could provide reasonable accuracy and precision for UAV remote-sensing missions. Strong correlation (R^2^ = 0.99) was consistently observed between UAV reflectance estimates based on radiometrically calibrated mosaics and actual ground reflectance. Average reflectance errors were less than 3% for all bands and generally less than 1% for the RGB bands. Average height error was within 10 cm for the lower and upper layers of the validation GCPs. The GCP system detailed in this article has excellent potential for multiple referencing purposes to reduce remote-sensing error and for automatic real-time communication to maximize efficiency.

## Figures and Tables

**Table 1:** Format of the GCP query instruction.

Start Marker	Device ID	Instruction ID	End Marker	CRC16 Check
2 bytes	1 byte	1 byte	1 byte	2 bytes

**Table 2:** Format of the global positioning system (GPS) data package of the requested GCP.

Start Marker	Device ID	Length	Long Left	Lat Left	Long Right	Lat Right	Alt Left	Alt Right	CRC 16
2 bytes	1 byte	1 byte	4 bytes	4 bytes	4 bytes	4 bytes	2 bytes	2 bytes	2 bytes

**Table 3:** Root mean square, standard deviation, and average error results measured for horizontal position, vertical position, and direction of the developed GCPs in the field.

	RMSE	STDV	AVG
Horizontal position	3.3 cm	2.1 cm	2.3 cm
Vertical position	4.6 cm	2.8 cm	4.2 deg
Direction	1.9 deg	0.5 deg	1.9 deg

**Table 4:** Format of the GPS data of the GCPs recorded in a txt file on the UAV.

GCP No.	Latitude_L (deg)	Longitude_L (deg)	Latitude_R (deg)	Longitude_R (deg)	Elevation (m)	Direction (deg)
1	30.530725	−96.427479	30.530734	−96.427497	39.95	121.39
2	30.529293	−96.428584	30.529280	−96.428597	39.30	40.10
3	30.532036	−96.428840	30.532049	−96.428853	38.55	138.40
4	30.533206	−96.430037	30.533219	−96.430051	39.27	136.48
5	30.533251	−96.432460	30.533264	−96.432446	38.93	222.65
6	30.535868	−96.429613	30.535881	−96.429599	39.60	221.58
7	30.536026	−96.427197	30.536014	−96.427183	38.45	316.52

**Table 5:** Radiometric calibration coefficients of the multispectral camera.

Band	K (Slope)	C (Intercept)	R^2^
475 nm	0.001334	−3.423	0.9906
560 nm	0.001192	−4.849	0.9924
668 nm	0.001434	−4.790	0.9943
717 nm	0.001082	−14.18	0.9849
840 nm	0.002539	−20.72	0.9622

**Table 6:** Average height errors for lower and upper GCP layers on each color tile.

Color Tile	GCP Layer	Height Error
Light gray	Upper	8.04 cm
	Lower	9.08 cm
Medium gray	Upper	8.47 cm
	Lower	9.67 cm
Dark gray	Upper	7. 95 cm
	Lower	9.86 cm
